# Caspar SUMOylation regulates *Drosophila* lifespan

**DOI:** 10.17912/micropub.biology.000288

**Published:** 2020-08-02

**Authors:** Bhagyashree Kaduskar, Deepti Trivedi, Girish S Ratnaparkhi

**Affiliations:** 1 Indian Institute of Science Education & Research, Pune, 411008 INDIA; 2 Fly Facility, National Centre for Biological Sciences (NCBS), TIFR, Bangalore 560065 INDIA

**Figure 1:**
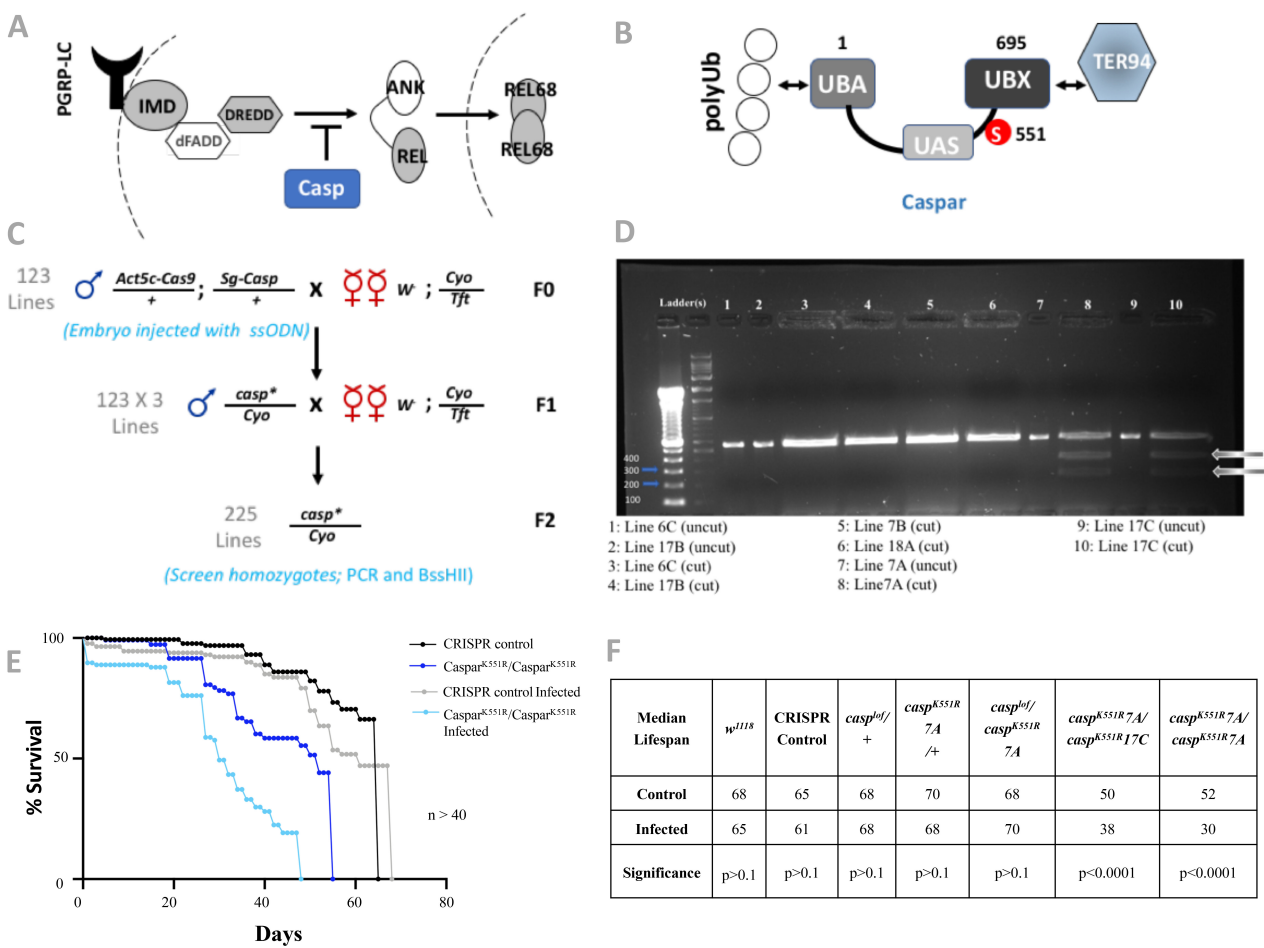
**Generation and characterization of a *casp^K551R^* transgenic fly by CRISPR/Cas9 genome editing.** **(A)** *Casp is a negative regulator of IMD/NFkB signaling*. Casp negatively regulates DREDD activity. DREDD is the protease responsible for cleavage/maturation of Relish (Rel), in response to IMD signaling and leads to the subsequent release of its N-terminal fragment Rel68, which travels to the nucleus to activate transcription. **(B)** *Domain structure of Casp*. Casp is orthologous to mammalian Fas associated factor 1 (FAF1) and contains an N-terminal Ubiquitin interaction domain (UBA), a ubiquitin-like (UAS) self-association domain and a C-terminal Ubiquitin regulatory domain (UBX). UBA domains are known to interact with Ubiquitin (Ub) and polyUb-chains (connected circles) while UBX binds specifically to the Transitional-ER-ATPase 94 (TER94; also known as valsolin containing protein -VCP or p97), which is member of the degradative Ub-proteasomal recycling system (Tresse *et al.*, 2010). The site of Casp SUMOylation (K551) (Handu et. al., 2015) is marked in red on the figure. Double-headed arrows indicate non-covalent interactions between Casp and other proteins **(C)** *Generation of putative lines for screening*. 350 0-3 hour old embryos expressing *Act5c-Cas9* along with the *gRNA* were injected with a 100 bp donor single-stranded oligonucleotide (ssODN). Adult males (n=123, one male per vial) that developed from these embryos were crossed to II^nd^ chromosome balancer females as a first step to stabilize putative mutant lines. Next, individual single parent crosses were set up to generate homozygous lines that were used to screen for mutants. **(D)** Screening for the casp^K551R^ mutant. PCR primers were designed to amplify 566 bp region spanning the site of mutation within the *casp* locus. A unique BssHII restriction site was engineered in the ssODN, allowing the successful genomic integration to be identified by a simple restriction digest. Representative PCRs from 225 lines confirm that two lines, 7A and 17C incorporated the ssODN by homologous recombination. Animals where the wild-type genome was retained (6C, 17B, 7B & 18A) were resistant to BssHII digestion and could be used as CRISPR controls. For the two positives lines 7A and 17C, two digestion bands of 350 bp and 216 bp were obtained (Arrows, lanes 8 & 10), confirming successful genome editing. This was further confirmed by DNA sequencing of the *casp* genomic region. **(E)** Survival plot for *casp^K551R^* transgenic lines for both uninfected and infected. The homozygous *casp^K551R^* mutant lines show a shortened lifespan. This lifespan is further shortened when animals are infected with gram-negative *E. coli*. The data were analyzed using Log-rank test of trend **(F)** Tabulation of median lifespan for Casp SUMO mutants along with all relevant controls.Values for the Median lifespan extracted from survival data were plotted using GraphPad 8.

## Description

Conjugation of Small ubiquitin-like modifier (SUMO) to a protein substrate (also called SUMOylation) is achieved by a specialized cellular machinery that includes a SUMO maturing enzyme (Ulp1, Ubiquitin-like protease 1) which also doubles as a de-conjugase, heterodimeric activation enzymes (Aos1 & Uba2), a single E2 conjugase (Ubc9), and multiple E3 ligases (Hay, 2005). Loss of function *ubc9* mutants shows activated immune signaling (Chiu *et al.*, 2005; Paddibhatla *et al.*, 2010), with Toll signaling attenuated by SUMO conjugation (Bhaskar *et al.*, 2002; Chiu *et al.*, 2005; Paddibhatla *et al.*, 2010). Although multiple studies have confirmed the role of SUMOylation in the regulation of *Drosophila* innate immune signaling (Bhaskar *et al.*, 2000); Bhaskar *et al.*, 2002; Chiu *et al.*, 2005), only a handful of proteins have been identified as direct targets of SUMOylation. Validated direct targets include Dorsal (Bhaskar *et al.*, 2002), and IRD5 (Fukuyama *et al.*, 2013), critical proteins in the Toll/NFkB and IMD/NFkB cascades, respectively. Previously, to gain a more elaborate understanding of the SUMO conjugation in the immune response, we performed a quantitative proteomic analysis and identified proteins whose SUMOylation status is altered in response to crude LPS, which activates both Toll/NFkB and IMD/NFkB signaling in *Drosophila* macrophage-like S2 cells. This analysis identified ~700 SUMO targets of which 5% were known to have immune function (Handu *et al.*, 2015).

In *Drosophila*, the IMD/NFkB pathway (Fig. 1A) is activated primarily in response to gram-negative bacterial infections (Brennan & Anderson, 2004; Kaneko & Silverman, 2005; Lemaitre *et al.*, 1995). When the PGPRP receptors recognize a pathogenic signature, a series of activation events leads to DREDD-dependent cleavage of the NFkB transcription factor Relish (Rel). This leads to translocation of cleaved Rel (Rel68) from the cytoplasm to nucleus and expression of numerous defense genes (Stöven *et al.*, 2000). All immune signaling pathways need strong negative regulation in the absence of infection as chronic expression of defense genes is detrimental to the health of the organism. One such negative regulator, for IMD signaling, is Caspar (Casp) (Fig1A, B). Casp negatively regulates the cleavage of Rel, by regulating DREDD activity, and thus retains the transcription factor in the cytoplasm (Kim *et al.*, 2006). Casp is a SUMO target that changes its SUMOylation state with infection (Handu et. al., 2015). Using an *in bacto* SUMOylation assay (Nie *et al.*, 2009), we have earlier established that Casp is SUMOylated at K551 (Handu et. al., 2015). Based on the domain structure of Casp (Fig. 1B), it possibly functions as an adapter for the ER-associated degradation pathway, assisting in the targeting of unfolded proteins to the ubiquitin-proteasomal system, using its N and C terminal domains to connect ubiquitinated proteins to TER94 (Tresse *et al.*, 2010).

To better understand the SUMOylation-specific biological effects associated with a protein, it is imperative to study it when its endogenous expression patterns and concentrations are maintained. To achieve this, we used CRISPR-Cas9 based genome editing to generate *casp^K551R^* transgenic lines. CRISPR mutagenesis techniques involve injecting a mixture of *casp* *guide-RNA (gRNA)* and donor DNA (*ssODN*, single-stranded oligonucleotide with codon CGC instead of AAG) into Cas9-expressing transgenic flies. The sequences of the *gRNA* and *ssODN* used are as listed in Materials & Methods. We initially utilized this ‘direct-injection’ protocol on 500 embryos but failed to obtain mutants. One reason for the failure could be the low efficiency of recognition of the *casp* genomic sequences by the designed *gRNA*. A DRSC tool (https://fgr.hms.harvard.edu/crispr-efficiency) predicts a score of 5.8 for this gRNA, much lower than the recommended 7 or above for successful editing. For a *ssODN* based strategy to generate a site-directed mutant, the *gRNA* sequences chosen had to be at/near the site of mutation for efficient homologous recombination, which limited our ability to design an alternate gRNA. Hence, keeping the gRNA constant, we decide to increase efficiency by using the alternate but slower protocol of first generating a *U6.2-casp-gRNA* transgenic line based on the protocols of (Kondo & Ueda, 2013). Subsequently, we crossed the *U6.2-casp-gRNA* transgenic flies with Cas9-expressing flies and collected the F0 embryos. We injected the *ssODN* containing the *K551R* mutation in ~350 embryos. Only a small fraction of the adults that eclosed from these injected embryos (Fig 1C) were expected to have gained the mutation. We chose only male flies (n=123) and crossed each male to five w^1118^; Cyo/Tft females. In the F1 generation, from each numbered vial we took three independent males (e.g Labeled 1A, 1B, 1C for the first line) and generated a 123 X 3 = 369 stocks for screening. With no known visible phenotype, efficient screening of flies with the desired mutation was facilitated by the addition of a unique restriction site—BssHII—at the site of mutation during ssODN design. The genomic DNA flanking the site of mutation was PCR amplified and digested with BssHII to check for the presence of the mutation (Fig.1D). We found that detection of the BssHII cleavage site in PCR amplified genomic fragments was easier to score in homozygous flies than in balanced heterozygotes. After screening 225 individual lines, two independent *casp^K551R^* mutant lines were obtained, namely lines 7A and 17C (Fig. 1D). The two lines are homozygous viable, have no obvious morphological defects. The lines have the desired codon substitution and no additional mutations/lesions in the *casp* locus. Quantitative real-time PCR confirmed comparable mRNA expression for wild type (DCt = 5.087) and the generated mutants (DCt = 4.343).

The lifespan of the 7A and 17C lines was measured (Fig. 1E, F) and we found that at 25 °C, both the *casp^K551R^/casp^K551R^* lines had shorter lifespans than the controls (*w^1118^* or *casp^K551R^/+* or CRISPR control), with the change in median lifespan being shorter by 13-20 days, suggesting that Casp SUMOylation contributed to a normal lifespan. A previous study has shown that overexpression of Casp reduces the lifespan of flies post-infection (Kim *et al.*, 2006) linking the activity of Casp to lifespan. Further, *Escherichia coli* infection shortened the lifespan of the *casp^K551R^/casp^K551R ^*by an additional 12 days (Fig. 1E, F) with controls (*w^1118^* or *casp^K551R^*/+ or CRISPR control), being able to deal with the *E. coli* infection without significant change in median lifespan. Interestingly a the *casp^K551R^/casp^lof^* line behaved like control with a median lifespan of 70 days without and 68 days with an infection. In our hands, the *casp^lof^* line shows low levels (~5-10%) of Casp^wt^ expression when compared to w^1118^.

To the best of our knowledge, this is the first study involving the application of CRISPR mutagenesis to generate a protein variant that is SUMO conjugation resistant. It is not clear at this point if Casp^K551R^ is a stronger or weaker negative regulator of DREDD mediated Rel cleavage. If the mutant protein is a better negative regulator, it may lead to a slower and/or weaker IMD/NFkB immune response. If the allele is weaker negative regulator, it may lead to overactive IMD/NFkB signaling and subsequent inflammation. Either of the above possibilities would lead to a shorter lifespan and increased susceptibility to gram-negative bacteria. This exact mechanism remains to be uncovered.

All the data taken together suggest a need of a delicate balance between the number of non-SUMOylated and SUMOylated Casp moieties for a regulated immune response. FAF1, the mammalian homolog of Casp, also regulates NFkB signaling by regulating the levels and nuclear translocation of RelA (Menges *et al.*, 2009). Although FAF1 itself has not been demonstrated to be SUMOylated, it has SIM motifs and interacts with SUMOylated proteins indicating a critical role of SUMOylation in FAF1 regulated processes (Wang *et al.*, 2019). It will be worth investigating whether molecular mechanisms for both Casp and FAF1 in immune signaling are conserved by the evolutionary process.

## Methods

*Fly strains and maintenance:* All flies were maintained on standard cornmeal media at 25 °C*.* The *casp^lof^* line (11373) is a piggyBac insert in the *casp* locus and was procured from the Bloomington *Drosophila* Stock Centre (BDSC).

*Survival Analysis*. For survival assays, 2-day old males from each genotype were maintained on standard medium at 25 °C. For survival post infection, 100 flies were pricked with a 20-hour old culture of ampicillin-resistant *E. coli* (DH5α). Dead flies were removed every day and food vials were changed every alternate day. Surviving flies were scored till all the flies were dead. Gehan-Breslow-Wilcoxon and Log Rank tests were performed using GraphPad Prism 8.0 to analyze the data (Piper & Partridge, 2016).

*CRISPR cloning and mutagenesis: Sg2-Casp gRNA*, *TGATCAGGTGAAGGCAGAGCAGG* cloned in *pBFv-U6.2B*. This clone was injected in *y^1^, v^1^, phiC1 integrase; attp40* embryos and the progeny were scored for v^+^ marker. The integrase was removed and the transgenics lines made homozygous.

ssODN sequence:

TTGCCGCATCCTTAGCCATGTCGGCTTGCAAAGTTTCCTGATAGGCCATGTCCTGCTCTGCGCG CACCTGATCACGGGCAGCGCGTTCGTCCTCTTGCCG

*Screening and sequencing of mutants*: Genomic DNA was extracted from individual homozygous flies, one from each line. PCR primers, *casp-F CTGGGCAGCAATTGCGAAGT* and *casp-R: GTAGCACCTACCTGTAGGTTG* were used to amplify a 566 bp PCR product (Figure 1D). PCR products from lines that showed BssH1 digestion, as well as controls that did not show digestion were sent for sequencing. Lines 7A and 17C were confirmed to have the AAG to CGC mutation, while the control lines retained the wild-type codon (AAG). Next, the entire *casp* locus for lines 7A and 17C was sequenced and was found to be identical to controls, with the exception of the engineered CGC mutation. The engineered mutation has remained stable in both 17C and 7A lines to date (~1 .5 years). *casp* expression levels were tested using quantitative real-time PCR with primers, *casp* F- CAAGCGTTTCCAATGTCAGAG; *casp R –* CTTAGAGCCTCCACAAGATTCC in the 7A, 17C lines as well as CRISPR controls.
